# Spermatorrhœa: Its Causes, Symptoms, Results, and Treatment

**Published:** 1871-03

**Authors:** 


### Book Notices.

---

Spermatorrhœa: Its Causes, Symptoms, Results, and Treatment. By Roberts Bartholow, A.M., M.D. William Wood & Co., Publishers, New York; for sale by W. B. Keen & Cooke, Chicago.

This little work is too well-known to the profession to require any extended notice. In this third edition the material has been rearranged, and in large part rewritten, and an appendix of formulæ added.

